# Male rutting calls synchronize reproduction in Serengeti wildebeest

**DOI:** 10.1038/s41598-018-28307-y

**Published:** 2018-07-05

**Authors:** Justin M. Calabrese, Allison Moss Clay, Richard D. Estes, Katerina V. Thompson, Steven L. Monfort

**Affiliations:** 1 Smithsonian Conservation Biology Institute, National Zoological Park, 1500 Remount Road, Front Royal, VA 22630 USA; 2 College of Computer, Mathematical, and Natural Sciences, University of Maryland, College Park, MD 20742 USA

## Abstract

Tightly synchronized reproduction in vast wildebeest herds underpins the keystone role this iconic species plays in the Serengeti. However, despite decades of study, the proximate synchronizing mechanism remains unknown. Combining a season-long field experiment with simple stochastic process models, we show that females exposed to playback of male rutting vocalizations are over three times more synchronous in their expected time to mating than a control group isolated from all male stimuli. Additionally, predictions of both mating and calving synchrony based on the playback group were highly consistent with independent data on wildebeest mating and calving synchrony, while control-based predictions were inconsistent with the data. Taken together, our results provide the first experimental evidence that male rutting vocalizations alone could account for the highly synchronized reproduction observed in Serengeti wildebeest. Given anthropogenically driven losses in many areas, a mechanistic understanding of synchrony can highlight additional risks declining wildebeest populations may face.

## Introduction

Highly synchronized reproduction occurs in a broad range of taxa and is one of nature’s most remarkable spectacles, with effects that cascade through entire ecosystems. Examples include mast seeding of trees^1,2^, pulsed emergence of periodical cicadas^3^, synchronized hatching of sea turtles^4^, and mass calving of wildebeest^5^. In dense populations where newly born or emerged individuals are highly vulnerable to predation, such synchrony can substantially reduce per-capita mortality rates either by predator satiation, predator confusion, or a combination thereof^5,6^.

The western white-bearded wildebeest (*Connochaetes taurinus mearnsi*) is a keystone species of the Serengeti-Mara ecosystem^7,8^. With a population of approximately 1.25 million animals^9^, it outnumbers all other Serengeti herbivore species combined^10^. Through its super-abundance, the wildebeest has profound effects on vegetation structure, fire regimes, food-web structure, and the abundance of many other species including both herbivores and predators^10–13^.

Reproductive synchrony is a key ingredient in the wildebeest’s remarkable abundance^5,10^. It displays mating and calving peaks that result in the birth of nearly 500,000 calves within a roughly three week period^5^. The degree of synchrony in wildebeest far exceeds that predicted by climatic seasonality and resource availability, with wildebeest exhibiting much greater synchrony than closely related species living in the same environment^10^. Instead, this sharp calving peak is the cornerstone of a highly effective anti-predation strategy that functions through a combination of predator swamping and predator confusion^5,10,14^. However, despite decades of study, the proximate mechanism that synchronizes such a vast population remains elusive^10,15,16^.

Theory suggests that social signals among individuals can modulate reproductive synchrony in vertebrates, but the specific cues vary from case to case^6,17^. For wildebeest, any effective synchronizing cue must simultaneously be accessible to the majority of the ~500,000 females in the migratory population^8^. While chemical cues mediate reproductive synchrony in many species, they are unlikely to function as a synchronizing mechanism for wildebeest given that females do not investigate the urine of conspecifics or perform flehmen^18^. Male presence alone is also an unlikely trigger, as exposure to males appears largely invariable year round.

Auditory mating stimulii provide an alternative class of social cues and are known from many taxa including birds^19^, amphibians^20^, and to a lesser extent, ungulates^21,22^. For example, McComb^21^ found that, in red deer, vocalizations by individual males advanced ovulation timing in harem females, thus improving mating opportunities for harem-holding males. In wildebeest, calling by territorial bulls is common throughout the year, but vocalizations during the three-week rutting peak are expressed at a higher than normal tempo, and by virtue of the number of both territorial and bachelor males participating, the call volume is far greater than at other times of the year^18^. Here, we hypothesize that a chorus of multiple male vocalizations modulates female wildebeest ovarian function in a way that reduces both the average time to mating, and the variance in time to mating among females. We predict that this modulation will be sufficient to facilitate the observed degree of both mating and calving synchrony in wild wildebeest populations.

**Figure 1:**
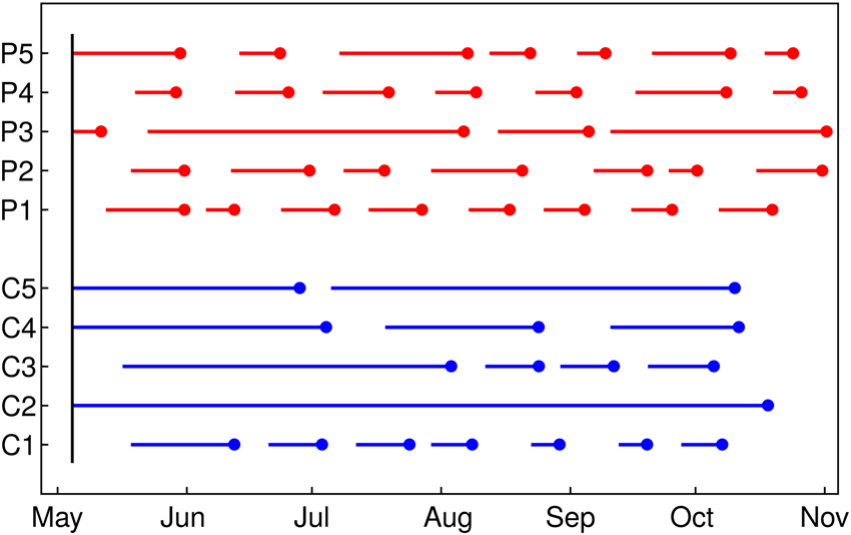
The individual time series of pre-ovulatory periods for each of the five Control (blue) and five Playback treatment (red) animals from the start of the experiment (vertical back bar) until 26 weeks thereafter. Individuals are organized vertically along the y-axis with each animal identified by an alphanumeric code. For example, the first Control individual is C1, while the last Playback individual is P5. The end of each pre-ovulatory period is highlighted with a point, as this is the time when a female can mate and become pregnant. There were 34 pre-ovulatory periods observed for the Playback group, and 17 observed for the Controls.

To test the effect of male rutting calls on reproductive synchrony, we combined a season-long field experiment with a stochastic process modeling approach. Females captured from the main, migratory Serengeti wildebeest population were first habituated to the presence of researchers and then were randomly assigned to one of three treatments. One group was completely isolated from male stimuli (Control, *N* = 5). A second group was exposed only to male vocalizations via playback of recorded rutting calls (Playback, *N* = 5). A third group was housed with an intact territorial bull and was also exposed to recorded rutting calls (Playback + Bull, *N* = 5). All groups were approximately 1 km away from the nearest neighboring group, with undulating terrain in-between in all cases, and thus were maintained in visual, auditory, and olfactory isolation from each other. Recordings were played continuously for three weeks to simulate the vocalizations females experience during the rut. Previously validated immunoassays for fecal progesterone metabolites were used to identify pre- and post-ovulatory periods^23^. Females were monitored individually for at least 26 weeks from the start of the experiment. This resulted in long time series of alternating pre- and post-ovulatory periods for each Playback and Control female (Fig. 1). In the Playback + Bull treatment, four females were fertilized shortly after the start of the experiment, while the fifth individual was determined (via oral examination, poor body condition, lethargy, and lack of appetite) to be of post-reproductive age and was consequently removed from the experiment^23^. The Playback and Control groups formed the basis of our analysis of the effects of vocalizations on synchrony. The Playback + Bull group was used only to justify a key assumption (see below) that allowed us to predict mating and calving synchrony from the Playback and Control results.

The transition from the pre- to post-ovulatory period marks the point in time (ovulation) when a female can become pregnant, and thus the duration of the pre-ovulatory period should largely govern the time to mating. Throughout the paper, we quantified synchrony as the shortest possible time for the focal event (e.g., ovulation, mating, or calving) to occur for 80% of individuals, denoted ${T}_{80}$. This metric is widely used in the literature^24^, has an intuitive interpretation, automatically captures the peak of the season, and can be easily estimated as the 80% highest density region^25^ of the focal time-to-event distribution. We used a non-parametric permutation test to formally compare synchrony between the Control and Playback groups. Noting that the pre-ovulatory period consists of a component whose duration is highly variable, and of a follicular phase, which is of relatively constant length for ungulates^26–28^, we then modeled pre-ovulatory period duration as the sum of a homogeneous Poisson process^29^ for the variable component and a constant for follicular phase. This results in pre-ovulatory period durations following a shifted exponential distribution, and we used these fitted distributions to summarize our experimental results and to test the ability of the Playback and Control results to predict both mating and calving synchrony in previously published, independent datasets.

## Results

**Figure 2:**
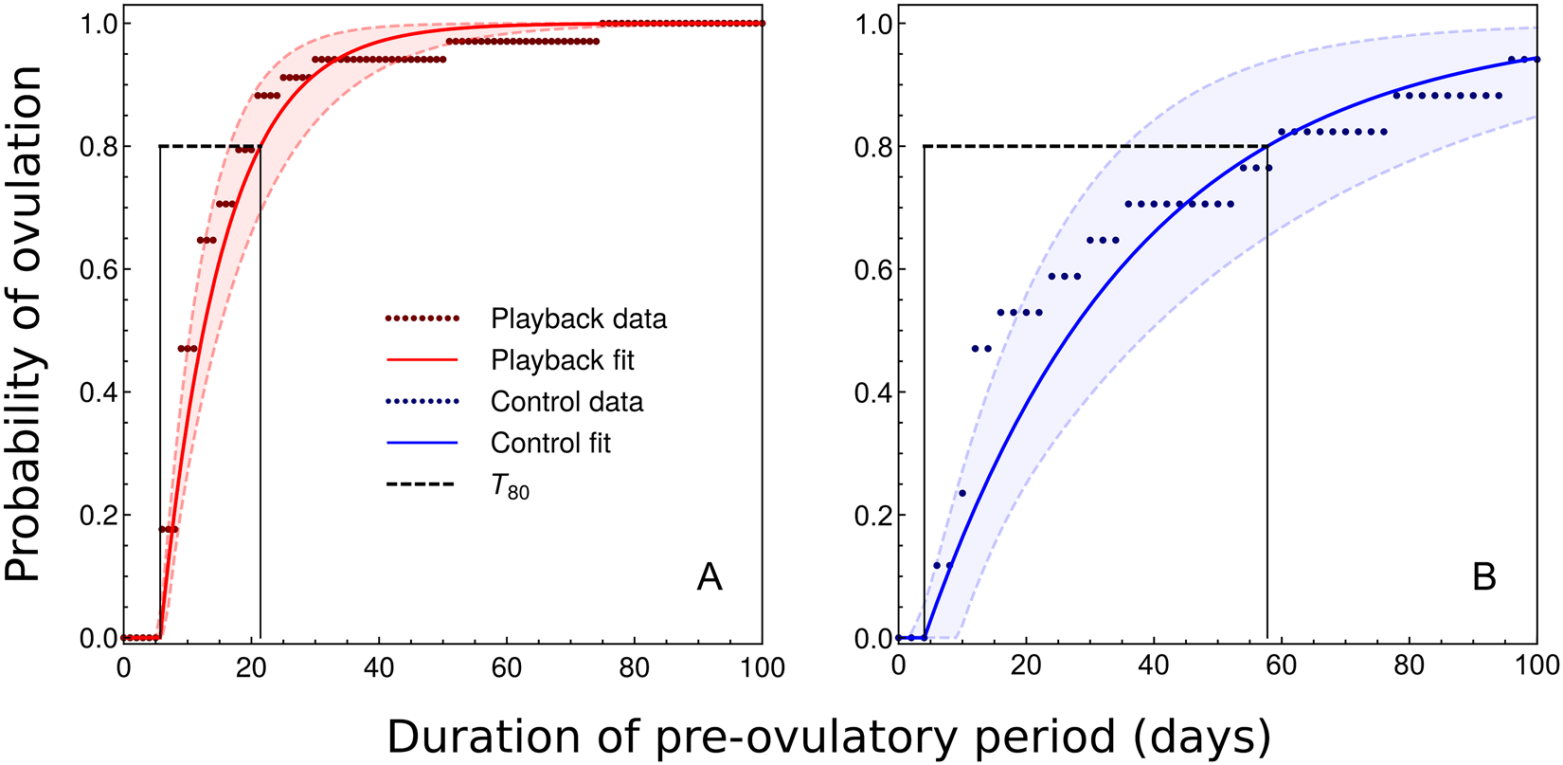
Cumulative probability of ovulation as a function of duration of the pre-ovulatory period. Panel A presents the data (dark red points) and fitted shifted exponential distribution (red curve) for the 34 pre-ovulatory period durations observed for the Playback group, while panel B displays the data (17 pre-ovulatory period durations; dark blue points) and fitted model (blue curve) for the Controls. The matching-color shaded regions and dashed curves delimit the 95% confidence intervals associated with each model fit. Horizontal dashed lines show the ${T}_{80}$ synchrony metric, which is the shortest possible interval that contains 80% of the ovulation events. Note that the x- and y-axis ranges are held constant across panels.

The difference between treatments in the time to ovulation is visually apparent in the data (Fig. 1), and a non-parametric permutation test confirmed that this apparent difference was indeed statistically significant (*p* = 0.01). Figure 2 shows the fitted shifted exponential models against the data for both the Playback and Control treatments, with the steeper slope of the Playback group indicating both shorter and less variable pre-ovulatory periods than in the Controls. The estimated ${T}_{80}$ values were 15.70 days (95% CI: 10.81–21.57) and 53.72 days (95% CI: 30.70–83.30) for the Playback and Control groups, respectively, indicating far greater synchrony for the Playback group (Fig. 2). All else equal, these results imply that a population exhibiting the Playback group cycling rate would mate 3.42 (95% CI: 1.80–6.12) times more synchronously than a population exhibiting the Control rate.

**Figure 3:**
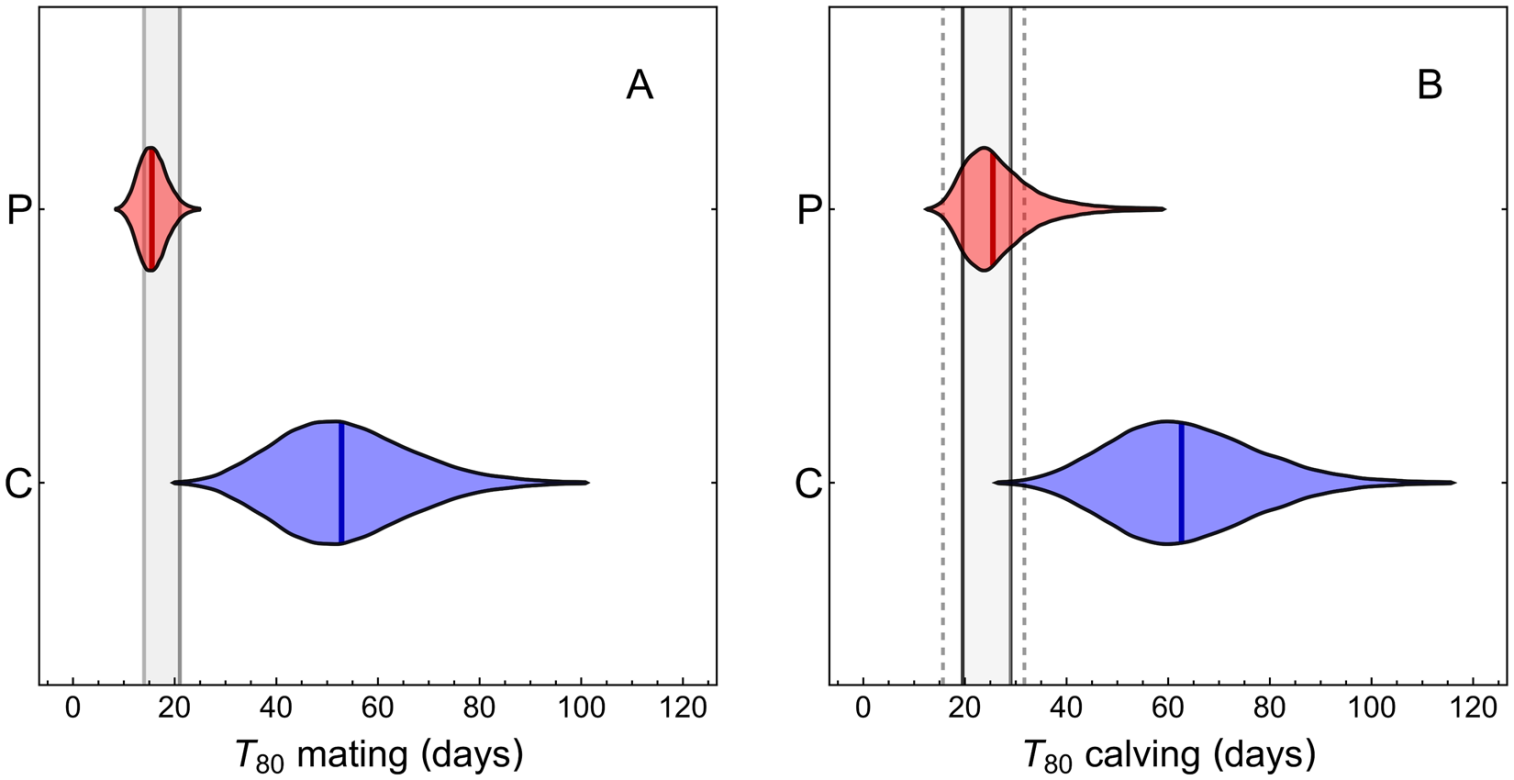
Violin plots displaying the bootstrapped prediction distributions of predicted ${T}_{80}$ synchrony values from the Playback (red) and Control (blue) groups. Color-matching vertical lines within each distribution show the median predicted value. (**A**) Predicted ${T}_{80}$ mating values for both groups compared to the range of empirical observations (vertical black lines and gray shaded region) of ${T}_{80}$ mating. (**B**) Predicted ${T}_{80}$ calving values for both groups compared to both the range of empirical point estimates (vertical black lines and gray shaded region) of ${T}_{80}$ calving, and to the outermost 95% confidence limits (vertical dashed lines) on the point estimates. The outermost limits are defined as the lower 95% CI limit on the smaller of the two ${T}_{80}$ calving estimates, and the upper 95% CI limit on the larger of the two estimates.

Based on post-mortem data, Watson^30^ reported that 80% of females mated within a two to three week window during the peak of the rut. To predict mating synchrony from our experimental results, we assumed that adult females in large herds are not mate-limited during the rut, which is consistent with field observations^9,10^, and that mating takes place during a short periovulatory interval. This later assumption is justified by our Playback + Bull treatment, in which the three healthy adult females mated and became pregnant around the time of their first ovulation in the presence of the bull, while the fourth female, a yearling, was fertilized on her second cycle. These experimental results are consistent with data on female mating success observed across 10 breeding seasons in the Serengeti, in which adult females have higher reproductive success (81–100%) than yearling females (4–80%)^7,31^. Under these assumptions, the time to mating is roughly equivalent to the time to ovulation, and we used parametric bootstrapping to generate a distribution of predicted ${T}_{80}$ mating values for each treatment from the fitted time-to-ovulation models (Fig. 3A). The median predicted ${T}_{80}$ mating value for the Playback group was 15.55 days and was within the range of the empirical estimates (Fig. 3A), as were 68.55% of the predicted ${T}_{80}$ mating values. In stark contrast, the predicted ${T}_{80}$ mating values for the Controls were much less synchronous than the data, with the median prediction (52.60 days) falling far outside the empirically observed range (Fig. 3A). Additionally, only 0.09% of the Control-based predicted ${T}_{80}$ mating values fell within the empirically observed range of 14–21 days (Fig. 3A).

Data from Estes^5^ showed calving synchrony ranging from ${T}_{80}$ = 19.56 days (95% CI: 15.67, 23.45) for large aggregations in 1973 to ${T}_{80}$ = 28.99 days (95% CI: 26.27, 31.70) for large aggregations in 1965. To go from our mating predictions to calving predictions, we further assume that wildebeest gestation periods are normally distributed with a standard deviation $(\hat{\sigma }=5.74)$ estimated from a scaling relationship across 41 mammal species^32^. We then used parametric bootstrapping of the fitted models plus normally distributed gestation periods to propagate uncertainty in parameter estimates into predicted ${T}_{80}$ calving values, and compared these predictions to the empirical estimates. As for the mating predictions, calving predictions based on the Playback group agreed well with the empirical estimates, with both the median prediction (25.46 days) and 57.35% of predicted values falling within the range of the empirical point estimates (Fig. 3B), and 77.91% of the predicted values falling within the outermost 95% confidence interval limits on the point estimates (Fig. 3B). In contrast, the Control-based predictions were highly inconsistent with the data, with the median (62.44 days) falling well outside the range of the empirical estimates, and only 0.15% and 0.36% of predictions falling within the ranges of the empirical point estimates, and outermost confidence intervals, respectively (Fig. 3B).

## Discussion

The annual phenomenon of birth synchrony among hundreds of thousands of Serengeti wildebeest is one of nature’s great wonders. Amazingly, despite more than 50 years of behavioral ecology research, little is known about the mechanisms controlling reproduction in this iconic species. Our novel approach permitted us to test the ability of male rutting vocalizations to synchronize reproduction in wildebeest maintained under controlled conditions within their native range, and to make out-of-sample predictions on both mating and calving synchrony that were validated by comparison to independent data.

Our findings represent the first experimental evidence of a synchronizing mechanism in the Serengeti wildebeest, and the first documentation of male rutting calls as a mechanism for synchronizing reproduction across a large ungulate population. Females exposed to rutting calls initiated cycles at over three times the rate of Control individuals and were consequently significantly more synchronous in their expected time to mating. While male presence^21,33–39^ and/or olfactory cues^39–42^ have been demonstrated to be an important modulator of female reproductive function in many mammal species, our results suggest that male vocalizations alone could be sufficient to account for the empirically observed reproductive synchrony in vast wildebeest herds.

While our experiment provided clear results, tight governmental controls on animal-based research protocols combined with the logistical difficulty of conducting the study under field conditions ultimately restricted our study to *N* = 5 females per treatment. Caution is therefore warranted in interpreting our results. Given that, our out-of-sample predictive analyses provide a critical check on the representativeness of the results we obtained. Specifically, the synchrony of both mating and calving predicted from the Playback group was highly consistent with independent, previously published empirical data. In clear contrast, predictions based on the Control group were far less synchronous than their empirically observed counterparts, with <1% of predicted ${T}_{80}$ values for both mating and calving falling within the observed range. That we obtained such a large difference between groups, with the nature of that difference being precisely that which allowed the Playback group, but not the Controls, to accurately predict independent mating and calving data, seems unlikely to have occurred by chance alone.

While our results demonstrate a clear role for male vocalizations, female synchrony could be achieved either by females responding independently to male calls or by females responding both to male calls and to changes in the hormonal status or behaviour of other females. Our experiment was not designed to tease apart these two candidate mechanisms, but there is sufficient variation among control group females to cast doubt on the latter hypothesis. Specifically, female C1 cycled at a rate that was comparable to Playback group individuals, but this did not induce the other control females in close contact with C1 to begin cycling at a similar rate. Furthermore, the chemical cues associated with reproduction in ungulates tend to be low volatility compounds carried in urine and secretions that are detected by the accessory olfactory system when females perform flehmen^43^. This type of chemical communication is unlikely to underpin wildebeest synchrony as it is: (1) short-ranged, (2) slow to propagate given the time needed for a receiving female to start producing new signal, and (3) relies on flemen, which wildebeest females do not perform^18^.

Watson^30^ originally proposed that reproductive synchrony in wildebeest might occur because early, “silent” ovulatory cycles in some females stimulate both the onset of normal ovulatory cycles in other females and the intensification of male-male competition, which ultimately leads to males mating with females regardless of the females’ degree of receptivity^10,30^. Given that chorusing is an indicator of competition, our results support the idea that male-male competition could be a key driver of synchrony in wildebeest. In contrast, our results cast some doubt on the hypothesis that female-female induction of ovulation plays an important role in achieving reproductive synchrony. However, the extent to which silently ovulating females might drive the intensification of male-male competition, and whether or not forced copulation of silently ovulating females is a significant component of reproductive synchrony, both remain topics for future studies. Additionally, Sinclair^44^ has argued that wildebeest reproductive synchrony is related to lunar cycles. To our knowledge, no physiological basis for such a mechanism is known for any mammal, and for wildebeest, no studies have provided additional evidence for the lunar cycle hypothesis beyond Sinclair’s^44^ original correlative results. Given our results, it seems unnecessary to invoke lunar entrainment to account for female synchrony.

The key ecosystem services provided by wildebeest in the Serengeti hinge critically on the species’ super-abundance^10,13^. By increasing per-capita survival rates on vulnerable neonates, reproductive synchrony is a core component in facilitating the high abundance of migratory wildebeest. However, the efficacy of this anti-predation strategy is strongly density dependent, with large herds experiencing substantially higher juvenile survivorship than small herds (84% vs. 51%) under the same conditions^5^. This type of positive density dependency can lead to accelerating losses as a population declines, which is often referred to as an Allee effect^45–47^. Whether or not this *component* Allee effect on juvenile survival translates into a *demographic* Allee effect, whereby the overall population growth rate decreases with declining density, is unknown for wildebeest.

Wildebeest are declining throughout much of their range^9,48–50^. The extent to which synchrony itself decreases with decreasing male density, and whether it does so gradually or abruptly, is currently unknown. However, the possibility of a positive feedback loop where both synchrony *per se*, as well as the anti-predation efficacy of a given amount of synchrony, decrease in concert in declining populations is troubling. Studies targeting the relationship between male density, chorusing intensity, and reproductive synchrony could therefore shed critical light on the extent to which such a vicious cycle is realized.

## Methods

### Study site

The study area is located within the Grumeti Game Reserve, in the Western part of the Serengeti ecosystem (33°30′ to 34°E and 1°30′ to 2°30′S). The Grumeti Game Reserve adjoins the corridor of Serengeti National Park north of the Grumeti River, and is within the natural range of the western white-bearded wildebeest.

### Animals, captures, and husbandry

This research was approved by the National Zoological Park’s Institutional Animal Care and Use Committee and permitted by Tanzanian Wildlife Research Institute and Tanzanian Commission on Science and Technology. All experiments were performed in accordance with the guidelines and regulations set forth by these agencies. Fifteen female wildebeest were captured from the main, migratory wildebeest population as it passed through the Grumeti Game Reserve. Animals were darted from a vehicle using capture darts containing etorphine (2–3 mg/animal) combined with xylazine (50–70 mg) administered i.m., according to anesthetic protocols that have been used successfully by Serengeti National Park veterinarians. The capture team was led by the Tanzanian Wildlife Research Institute (TAWIRI) veterinarian. Anesthetized animals were fitted with plastic ear tags, and transported by truck to the study site.

The cows were initially held in a 30 × 60 m boma that was contained within a large 500 × 500 m (25 ha) fenced enclosure. All individuals were exposed to natural fluctuations in climate and photoperiod, and allowed to graze on the natural growing grasses. Grazing was supplemented as needed with grasses cut from suitable neighboring habitat as well as a feed mix composed of maize meal, cottonseed cake, and salt. After successful habituation, the animals were released from the boma into the larger enclosure. The cows were left in the 25 ha enclosure throughout the calving season and until the youngest calf was two weeks old. At the end of March 2003, all animals were anesthetized and transported to one of three new enclosures, each 100 × 100 m. Adults and juveniles were randomly distributed amongst the three groups, and calves remained with their mothers.

The research camp and study facilities were constructed from scratch in a remote location with all work done manually and most transportation by foot or bicycle. Electricity was provided by a propane generator and was reserved for the use of lab equipment, freezing of fecal samples, and recharging the batteries used to run the playback equipment. The project required a crew of approximately 30 local workers (for fence construction/maintenance, cutting of supplemental grass feed, transporting water, guarding the study animals from predators, fecal sample collection, etc.) for the 2+ years of the field study. The logistical effort required, as well as the tight governmental controls on the use of wildlife for experimental research, limited the available sample size and disallowed any follow-up studies.

### Recording playback system

Two of the three groups (Playback and Playback + Bull) were exposed to recorded male vocalizations, while a third group was completely isolated from male stimuli and served as a control. All three groups were maintained in complete isolation from one another and from any external male cues. A compilation of recordings made during various ruts over the past 40 years was played over game caller speaker systems (Cabela’s Black Electronic Game Caller; Cabela’s, Sidney, Nebraska). To prevent habituation, the order of recordings was randomized and speaker locations were rotated daily to alternate the direction from which the sound emanated.

The recordings were played continuously (24 hours/day) for three weeks (beginning the morning of May 5) to simulate the vocalizations heard during the rut. This timing exposed the cows to the vocal cue within their normal breeding season, but several weeks earlier than the expected breeding peak in the migratory population. We anticipated that environmental factors would allow the animals to meet threshold nutritional requirements at this time, but that inducing ovarian activity prior to the migratory population would allow us to distinguish between potentially confounding environmental effects and those truly resulting from exposure to male vocalizations. An intact bull was introduced into the Playback + Bull group the night before the recordings were initiated (May 4). The male was a territorial bull from a nearby resident population (Kirawira area) captured and translocated using the same protocols employed for the females.

### Fecal sample collection and hormone extraction

Fecal samples were collected from each cow a minimum of once every three days by following the movements of the herd within their enclosures. Fecal extractions were conducted on site in a field laboratory; hormones were assessed in diluted fecal extracts, and pre- and post-ovulatory periods of ovarian cycles were identified as described previously^23^.

### Hypothesis and physiological assumptions

By quantifying fecal progesterone metabolites, we are able subdivide a female’s ovarian cycle into pre-ovulatory and post-ovulatory periods (Fig. S1). The transition between the pre-ovulatory and post-ovulatory periods (=ovulation) marks the point in time when a female can mate and become pregnant.

We hypothesize that male rutting vocalizations modulate ovarian cyclicity in a way that ultimately reduces the among-female duration and variability of the pre-ovulatory period. To test this hypothesis, we construct a simple stochastic process model of ovarian cycling in female wildebeest and then derive associated statistical measures from it. We assume that the pre-ovulatory period can be subdivided into a component that is highly variable in duration among individuals, and a follicular phase component that is constant in duration for all females within a treatment. These assumptions set a lower-limit (i.e., the duration of folicular phase) to the amount of time it takes a female to transition from the pre- to post-ovulatory period upon exposure to male rutting vocalizations.

These assumptions are reasonable as: (1) follicular phase durations in bovids are known to be roughly constant across both individuals and species, and to last approximately one week (e.g., follicular phase durations of sable antelope, scimitar-horned oryx, and addax as inferred from endocrine assays are 8.8d^26^, 5.5d^27^, and 5.1d^28^, respectively), and (2) some individuals in our experiment (e.g. four out of five in the Control group, and one out of five in the Playback group) had pre-ovulatory periods that lasted >30d, suggesting the non-follicular phase component must be highly variable among individuals.

Based on these considerations, we omitted three observations (one from the Control group and two from the Playback group) from females that entered the post-ovulatory period within three days of the start of the experiment. These females were likely already in follicular phase when the experiment began, and thus were unlikely to have been influenced by the presence (or absence) of male rutting vocalizations. We note that this omission is *conservative*, as including these observations in the analysis further strengthens the differences between the Playback and Control groups.

### Synchrony metric and hypothesis test

Synchrony is frequently described in the literature in terms of the time it takes a given percentage of the population to complete a focal activity^24^. This metric is usually tabulated during the peak of the season, when the occurrence rate of the focal activity is highest (e.g. see^5^). This implies that the time taken to reach the specified percentage is the shortest possible time in which this could occur for the focal dataset. To see this, consider that at any time outside of the seasonal peak, the occurrence rate of the focal activity must be lower, and it will thus take longer to reach the specified percentage.

Fortunately, this type of synchrony metric has a formal statistical definition. Given a probability distribution of the times taken to reach a particular event (e.g. ovulation, mating, calving), this metric is this highest density region (HDR)^25^ of that distribution that contains the specified percentage of events. As the HDR is, by definition^25^, the shortest possible interval that contains the specified percentage of events, it automatically zeros-in on the peak time of season. This metric can be calculated for any time-to-event distribution, and thus we present all of our results on synchrony, whether for ovulation, mating, or calving, in terms of the HDR. By convention in the synchrony literature, we choose 80% as the target percentage and refer to this metric hereafter as ${T}_{80}$. We estimated ${T}_{80}$ using the hdrcde package^51^ for the R environment for statistical computing^52^.

While it is clear from Figs 1 and 2 that the Playback and Control groups differ substantially, it is useful to perform a formal hypothesis on the between-group difference in ${T}_{80}$ ovulation synchrony. Under the null hypothesis of equal synchrony, the difference $D={T}_{80}^{C}-{T}_{80}^{P}$ is 0, and the one-sided alternative hypothesis that corresponds to our working hypothesis that ${T}_{80}^{C} > {T}_{80}^{P}$ (i.e. it takes the Controls longer to reach 80% ovulation because they are less synchronous) is *D* > 0. We used a non-parametric permutation test to test our hypothesis^53^. Specifically, we randomly permuted group labels (“Control” or “Playback”) while preserving within-group sample sizes. For each permutation, we calculated *D*_perm_, and repeated this procedure 50,000 times to estimate the distribution of *D* under the null hypothesis. We then calculated the observed value of the test statistic, *D*_obs_, from the original data and estimated the one-sided *p*-value associated with our hypothesis as the proportion of *D*_perm_ values ≥ *D*_obs_^53^.

### Modeling approach

Given the above assumptions, each individual pre-ovulatory period can be modeled as the sum of a constant representing the follicular phase and a stochastic component representing the remainder of the pre-ovulatory period (Figs S1 and S2).

The stochastic component can then be modeled as a homogeneous Poisson process, which assumes that the transition from a pre- to post-ovulatory period occurs at a constant rate *λ*. The durations of the pre-ovulatory periods for a given individual will then follow a shifted exponential distribution with probability density function (PDF)^54^

1 $${f}_{X}(x;\lambda ,\tau )=\{\begin{array}{cc}\lambda {{\rm{e}}}^{-\lambda (x-\tau )} & x\ge \tau \\ 0 & x < \tau \end{array}$$

where *x* is the pre-ovulatory period duration in days, *τ* ≥ 0 is follicular phase duration in days, and *λ* is the per-day rate at which the stochastic component of the pre-ovulatory period ends. This distribution has mean *τ* + 1/*λ* and standard deviation 1/*λ*. The parameter *τ* is effectively a nuisance parameter, as its value does not directly affect the outcomes of the analyses that follow, but obtaining a good estimate of *λ*, on which the results critically hinge, requires also estimating *τ*.

As we are interested in the treatment-level effect of rutting vocalization playback on ovarian cyclicity, we pool the data across individuals within each treatment resulting in *N*_*P*_ = 34 and *N*_*C*_ = 17 pre-ovulatory period durations for the Playback and Control groups, respectively. Figure S2 illustrates how we go from individual time series of ovarian cycles to the treatment-level shifted exponential distribution of pre-ovulatory period durations.

Model parameters were estimated via the minimum variance unbiased (MVU) estimators defined in^54^. Unless otherwise noted, all analyses were performed with Mathematica 10.0^55^. The MVU estimates for the Playback group were ${\hat{\lambda }}_{P}=0.10\,(95 \% \,{\rm{CI}}:0.08,0.15)$ and ${\hat{\tau }}_{P}=5.71\,(95 \% \,{\rm{CI}}:5.39,6.48)$, while those for the Control group were ${\hat{\lambda }}_{C}=0.03$ (95% CI: 0.02, 0.05) and ${\hat{\tau }}_{C}=4.03\,(95 \% \,{\rm{CI}}:1.62,9.25)$.

### Calving synchrony predictions

We have shown a statistically significant difference in synchrony between the Playback and Control groups in our experiment. We now demonstrate that the observed degree of synchrony in the Playback group can account for calving synchrony in the wild, while the synchrony of the Control group can not. To do this, we note that the calving (birth) time *B* is a random variable defined as

2 $$B=M+G$$

where *M* and *G* are the time to mating and the gestation duration, respectively. Under the above-described assumptions, our experiment provides estimates of *M* for both the Control and Playback groups. Gestation periods of mammals are frequently symmetric and well described by the normal distribution^56–61^, and consequently, we assume gestation periods are normally distributed with mean *μ* and standard deviation *σ*. The distribution of *B* in eqn 2 can be obtained by convolving the PDF of the normal distribution for *G* and the PDF of the shifted exponential distribution (eqn 1) for *M*, which results in calving times following a shifted version of the ex-Gaussian distribution^62^ with PDF

3 $${f}_{B}(b;\tau ,\lambda ,\mu ,\sigma )=\frac{\lambda }{2}{e}^{\frac{1}{2}\lambda (\lambda {\sigma }^{2}+2\mu +2\tau -2b)}\,{\rm{Erfc}}\,(\frac{\lambda {\sigma }^{2}+\mu +\tau -b}{\sqrt{2}\sigma })$$

where ${\rm{Erfc}}(\bullet )$ is the complementary error function. The variance of the shifted ex-Gaussian distribution is

4 $${\rm{Var}}(B)={\rm{Var}}(M)+{\rm{Var}}(G)$$

5 $$=\frac{1}{{\lambda }^{2}}+{\sigma }^{2},$$

which demonstrates that the location parameters *μ* and *τ*, which only shift the distribution laterally in time, have no effect on the spread of the calving distribution, and therefore do not affect its synchrony.

We estimated *σ* via a scaling relationship obtained by regressing Log_2_(*σ*) against Log_2_(*μ*) on a dataset of gestation durations for 41 mammal species (Fig. S3)^32^. We used all species in the dataset except for primates and elephants, which Kiltie^32^ noted appeared to follow a very different relationship than everything else. We estimated the mean gestation duration for wildebeest (*μ* = 258 d) as the mid-point of the range reported in the literature (242 to 274d)^18,23,63^. We then obtained the prediction distribution of *σ* from the fitted regression model evaluated at a mean gestation length of 258d (Fig. S3).

For each of the Playback and Control groups, we generated a predicted calving distribution via parametric bootstrap by first drawing a random value of *λ* based on the experimental results (see above) and a random value of *σ* from the prediction distribution of the standard deviation in gestation. We then substituted these values into eqn 3 and calculated ${T}_{80}$ calving. Repeating this procedure 15,000 times for each group then produced predicted ${T}_{80}$ calving distributions for each treatment. Notice that the gestation component is the same for both the Playback and Control groups, so differences in the calving predictions arise from differences between the groups in the expected time to mating.

### Code availability

The Mathematica and R scripts used in this analysis will be provided by the authors upon request.

### Data availability

The data used in this study will be publicly available in the Dryad (datadryad.org) repository upon publication.

## Electronic supplementary material

Supplementary Figures
