# Supplementary material for: Male rutting calls synchronize reproduction in Serengeti wildebeest
Source: Sci Rep. 2018 Jul 5;8:10202. doi: 10.1038/s41598-018-28307-y (PMC6033926; doi:10.1038/s41598-018-28307-y)
Supplement: Supplementary file 1 — Supplementary Figures [file 41598_2018_28307_MOESM1_ESM.pdf]

Supplementary figures for: Male rutting calls synchronize reproduction in Serengeti wildebeest

Justin M. Calabrese,[1*†] Allison Moss Clay,[1*†] Richard D. Estes,[1]
Katerina V. Thompson,[2] Steven L. Monfort[1]

[1]Smithsonian Conservation Biology Institute, National Zoological Park
1500 Remount Road, Front Royal, VA 22630
[2]College of Computer, Mathematical and Physical Sciences
University of Maryland, College Park, MD 20742

[†]Co-corresponding authors: CalabreseJ@si.edu & amossvan@hotmail.com.
[*]Co-first authors, order of authorship is alphabetical.

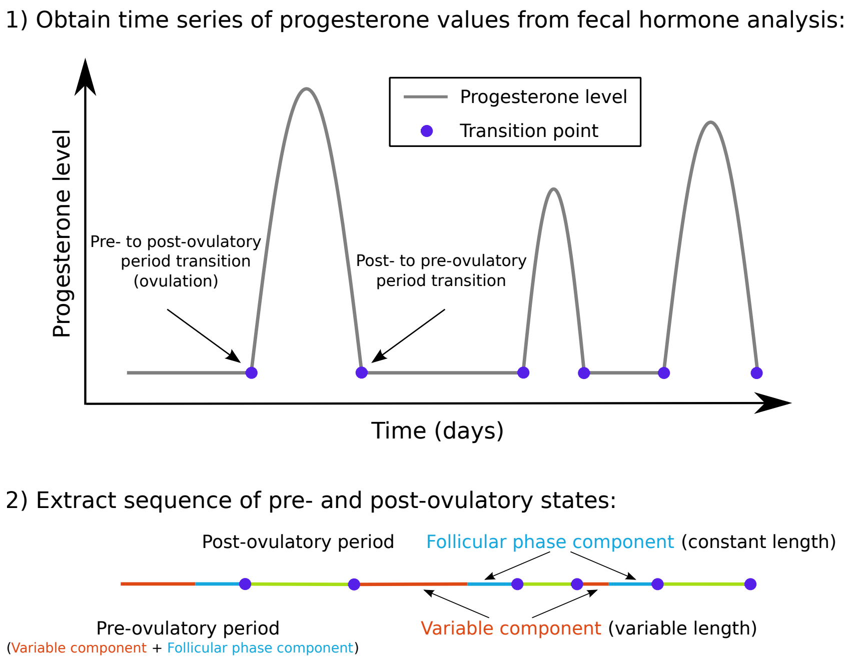

Figure S1: Schematic diagram of wildebeest endocrine data processing. Based on cyclical variation in progesterone levels in the time series of fecal progesterone metabolites, pre-ovulatory (light blue + orange; low progesterone) and post-ovulatory (green; high progesterone) periods can be distinguished. We then assume, based on available evidence, that the pre-ovulatory periods consist of two components: a follicular phase of constant length (light blue), and a component that varies in length (orange). Notice that the transitions in parts 1 and 2 of the figure are horizontally aligned.

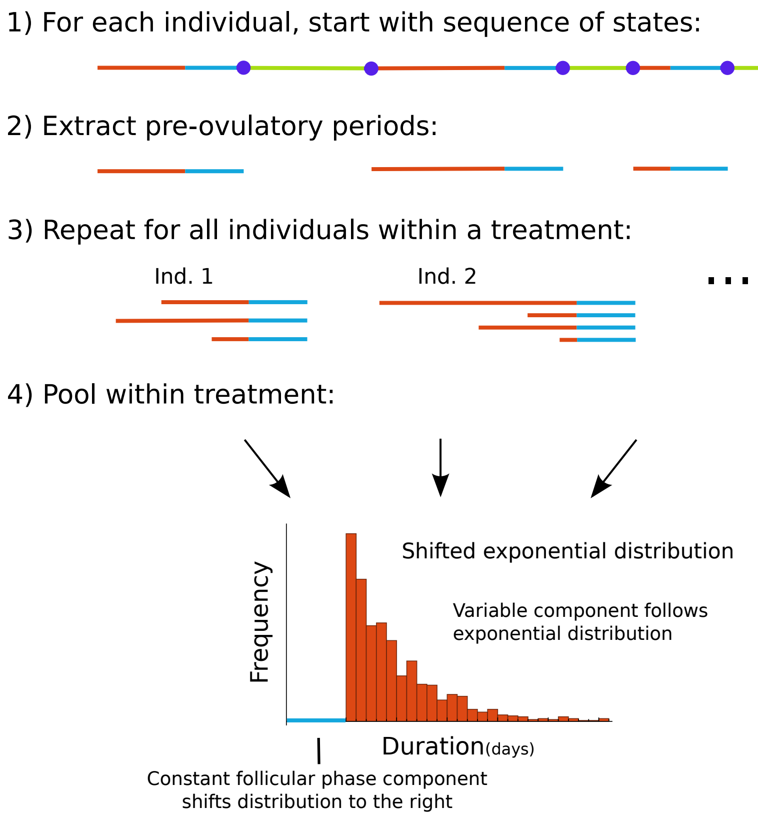

Figure S2: Schematic representation of the relationship between the underlying reproductive physiology and the shifted exponential model of pre-ovulatory period durations. Pre-ovulatory periods are extracted for each individual, and then pooled for all individuals within a treatment. The length of the constant follicular phase, τ, and the rate at which the variable component ends, λ, are then estimated from the pooled data for each treatment.

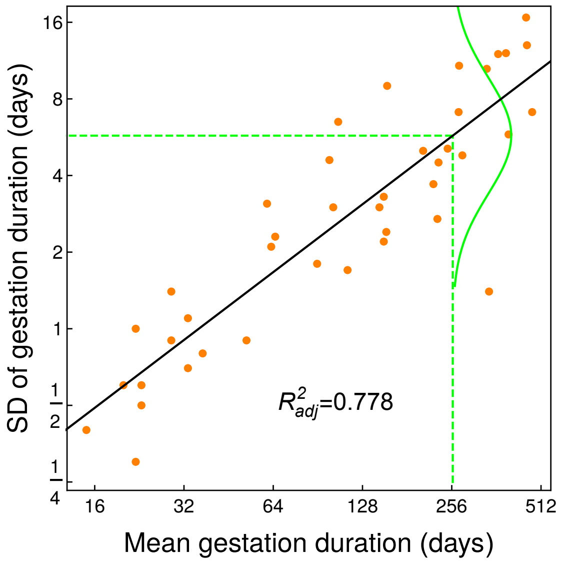

Figure S3: Regression relationship (black line) between the standard deviation, σ, and mean, μ, of gestation durations for 41 mammal species (orange points). The dashed green line shows the point estimate of σ at $\mu = 258$d, while the solid green curve shows the prediction distribution of σ centred on the point estimate. The prediction distribution was used, together with the experimental results, to predict calving synchrony.
